# Public and patient involvement in the development of an internet‐based guide for persistent somatic symptoms (GUIDE.PSS): A qualitative study on the needs of those affected

**DOI:** 10.1111/hex.13931

**Published:** 2023-12-07

**Authors:** Eirin Fränkl, Nele Hasenbank, Karin Dumröse, Bernd Löwe, Sebastian Kohlmann

**Affiliations:** ^1^ Department of Psychosomatic Medicine and Psychotherapy University Medical Centre Hamburg‐Eppendorf Hamburg Germany; ^2^ Patient Representative Hamburg Germany

**Keywords:** internet‐based intervention, intervention development, persistent somatic symptoms, public and patient involvement

## Abstract

**Background:**

Persistent somatic symptoms (PSS) frequently remain under‐treated in health care settings. Evidence‐based services that lead affected individuals to early guideline‐based care are currently missing. This study aimed to identify the needs of those affected concerning an internet‐based guide. The second aim was to evaluate public and patient involvement (PPI).

**Methods:**

Participants experiencing PSS for at least 6 months were recruited via hospitals, psychotherapeutic practices and self‐help organizations. Qualitative data were gathered via ideation discussions and prioritization tasks. Thematic analysis was conducted to gain insight on the needs of people with lived experiences. PPI was quantitatively evaluated with the Public and Patient Engagement Evaluation Tool.

**Results:**

A total of 12 individuals participated (eight females, ages 22–66 years, duration of symptoms 1–43 years). Participants wanted to feel more supported, validated, in control and engaged with managing their health. Content‐related preferences included education, self‐help, social support and contact addresses. The majority of participants (>90%) experienced their involvement as worthwhile.

**Conclusions:**

To the best of our knowledge, this is one of the first studies describing PPI in intervention development for PSS. The involvement was perceived as a valuable contribution to the development process.

**Patient or Public Contribution:**

Adults with lived experiences were involved at the level of collaboration through the establishment of a participatory research team (PRT) and at the level of consultation through a workshop series, including one of the coauthors. They were involved in developing and validating intervention material and checking data interpretation.

## 1 INTRODUCTION

Persistent somatic symptoms (PSS) refer to subjectively distressing somatic complaints that occur on most days for several months and are frequently accompanied by excessive health‐related concerns.^1^ Symptoms are heterogeneous and may include pain, cardiovascular, gastroenterological and neurological symptoms. These symptoms may or may not be associated with a known underlying medical condition and are often referred to as medically unexplained symptoms, functional disorders, somatoform disorders or somatic symptom disorders. PSS are described as a perceptual dysregulation, where processing and perception of bodily symptoms are disturbed.^2^ Regardless of their aetiology, PSS are highly prevalent in the general population^3^ and are associated with high mental and physical burden,^4^ as well as exorbitant health care utilization.^5^ While there is evidence‐based treatment available,^6^ there is a large gap from first onset to evidence‐based treatment, resulting in a long‐lasting disease burden.^7^ What is currently missing are services that guide affected individuals to evidence‐based care at an early stage.

Novel interventions are needed to address the existing treatment gap for PSS. Newer guidance for intervention development emphasizes the importance of involving stakeholders in intervention development.^8^ Public and patient involvement (PPI) initiatives have become of increasing importance within academic research. PPI is defined as ‘research being carried out “with” or “by” members of the public, rather than “to”, “about” or “for” them’.^9^ While people with lived experiences traditionally are viewed as passive research subjects, PPI strives to treat those affected as active research partners. Their involvement can range from contributing knowledge to actively managing the research process.^10^ PPI can inform both qualitative and quantitative research approaches. The anticipated benefits of PPI include making research more relevant, accessible and appropriate for the general public.^11^ Evidence indicates that PPI strengthens the quality of research^12^ and benefits both researchers and those affected.^13^

While PPI does not generate data, it provides the opportunity to take end users' perspective into account when developing novel interventions, thus ensuring acceptability and feasability.^8^ People with lived experiences can contribute to the development process in various ways, for example, by prioritizing relevant outcomes and sharing their perspective on design and content. For instance, PPI substantially changed the initial draft of a self‐harming behaviour intervention^14^ and contributed to the design of a feedback intervention for depression.^15^ Those affected also defined key outputs of a diabetes intervention^16^ and features of an intervention for increased physical activity in individuals with rheumatoid arthritis.^17^ There are previous studies investigating the needs of those affected by PSS concerning internet‐based interventions,^18^ but there is a lack of research on PPI during the development of novel interventions. PPI may be especially valuable within research on PSS, as those affected often are viewed as a burden in health care settings and feel their needs are not taken seriously.^19^ PPI provides the opportunity to incorporate views of people with lived experiences, thus tailoring the intervention to a previously unreached target population.

One particular challenge for the implementation of PPI is the lack of consistent reporting and evaluation.^20^ Considering the need for clearer guidance and understanding of how PPI contributes to mental health interventions, transparent reporting according to available frameworks is necessary. This will give further insights on how people with lived experience can be involved in the development process and how interventions can reach their target population.

To the best of our knowledge, this is the first study reporting PPI in the early development of an internet‐based guide for PSS (GUIDE.PSS). The first aim of this paper is to identify the needs of those affected by PSS for an internet‐based guide. The second aim is to evaluate the impact, process and outcome of PPI in intervention development.

## 2 METHODS

### 2.1 Study design

In this study the development of a prototype of an internet‐based guide for PSS was informed by qualitative methods and principles of PPI according to the guidelines provided by the National Institute for Health and Care Research.^9^ The prototype was developed between November 2022 and June 2023. This study was approved by the local Ethics committee at the Centre for Psychosocial Medicine (#LPEK‐0554). The project was registered at Open Science Framework.^21^

The development of the GUIDE.PSS prototype included literature review, workshops and a creative design phase involving a graphic agency. The development was based on the Integrate and Design phase of the IDEAS Framework,^22^ which describes the process of gathering insights from users and relevant theories followed by iteratively designing a prototype with user feedback. We applied suitable behaviour change techniques for online patient information material (e.g., Shaping Knowledge, Natural Consequences and Identity^23^) and followed recommendations made by current guidelines for PSS.^24^

### 2.2 PPI

People with lived experiences contributed to the designing and managing phase of the research cycle.^9^ This included developing a shared research agenda, prioritizing research questions and contributing to the development of the intervention material. PPI was implemented at the level of consultation through a series of four 3‐h workshops moderated by the junior researchers and principal investigator (see Section 2.2.1). In addition, PPI was realized at the level of collaboration through the establishment of a participatory research team (PRT) (see Section 2.2.2). Stakeholder discussions with representatives from a health insurance, a digital health agency and a self‐help organization contributed further. PPI was reported following the GRIPP2 reporting checklist.^25^

#### 2.2.1 Workshops

Individuals affected by PSS were involved in the development process by participating in four workshops (WS). Semi‐structured guides were defined beforehand, consisting of questions designed to explore participants' needs and preferences. The WS followed an iterative process, where each WS was adapted according to the previous ones. All WS were characterized by a democratic approach, where participants and researchers contributed equally. All ideas generated during the WS were discussed within the group to reach consensus. Participants' were involved in decision‐making processes on content, language and design of the prototype to ensure the acceptability and relevance of the developed material. In addition, they prioritized outcomes for the follow‐up evaluation of the guide. Qualitative methods were applied to gather data on the needs of those affected for an internet‐based guide. Participants shared their ideas in smaller groups of 3–4 individuals, followed by group discussions with everyone, to create an environment supporting equal participation. The WS were recorded with participants' consent.

In WS I the aim was to gain insight on participants' perspectives on barriers in current healthcare via brainstorming tasks. The second aim was to collect content‐related needs for an internet‐based guide in a mind map. Each task was followed by a moderated group discussion. Following WS I, the research team drafted a preliminary paper version of the prototype.

In WS II preferences on design, features and language were evaluated. Participants rated existing internet‐based information material on PSS^24^, ^26^ and the preliminary prototype. Participants' preferences concerning the use of numbers, case studies, figurative language, illustrations, bullet points, and the general tone were evaluated with a sample text. Based on the collected data from WS I and WS II, the graphic agency designed two dummy versions of the prototype.

The aim of WS III was to obtain feedback on the design and language of the dummy versions. The second aim was to define what associations the guide should evoke via moderated group discussion. Following WS III, the PRT and research team took part in a WS held by the graphic agency to discuss the colour scheme, fonts and illustrations. Based on this and data from WS III, the graphic agency adapted the design of the prototype. The research team drafted sample content based on WS I, WS II, WS III and existing patient information material on PSS.^22^

In WS IV, the objective was to obtain final feedback on the design of the prototype and language of the sample content. Participants received sample content on prevalence, aetiology, course of illness, treatment recommendations and self‐help strategies for PSS from the prototype as well as existing patient information material. They were asked to rate and compare the samples. WS IV was completed with a final feedback round.

#### 2.2.2 Participatory research team

Three individuals with PSS (two female, one male) were involved in the PRT to enable an ongoing partnership between those affected and researchers. The PRT participated in the WS series and in an additional four meetings to collaborate on the intervention development process. All PRT meetings were characterized by a democratic approach with shared decision‐making processes. They were involved in discussions regarding the research agenda, workshop guides, intervention material and the drafting of the manuscript. Member checking was applied to ensure the researchers' interpretation of the data was accurate and incorporated in the prototype according to their perspective.

In the first PRT meeting, PRT members were informed about the aim and methods of the study. The principal investigator gave a brief introduction on fundamentals of psychological research, qualitative and quantitative research methods, phases of the research cycle and intervention development. PRT members received information on the principles of PPI and they made an informed decision on what roles and assignments they wanted to complete during the project.

In the second PRT meeting preliminary results from WS I were discussed. Members of the PRT commented on conclusions they had drawn from WS I concerning perceived barriers in healthcare and content‐related needs. The workshop guide for WS II was discussed to reach consensus on the aims of WS II.

In the third meeting, the PRT participated in a meeting held by the external graphic agency. All members of the PRT were equally involved in decisions made concerning the design of the GUIDE.PSS prototype, including colour scheme, fonts and illustrations.

In the fourth PRT meeting, drafts of the prototype were discussed. The PRT checked the qualitative data interpretation and how the generated results were incorporated in the prototypes. A first version of the manuscript was presented and PRT members decided if they wanted to collaborate on the manuscript. The meeting was concluded with a final feedback round.

### 2.3 Sampling

The participants were purposefully selected to ensure a diverse sample. People were eligible for participation if they reported experiencing PSS for at least 6 months and were older than 18 years. Participants with an acute physical or mental illness requiring immediate treatment, substance abuse disorders, or psychotic illnesses were excluded. Eligible participants at the University Medical Centre Hamburg Eppendorf, who had previously agreed to be contacted for study purposes, were recruited via telephone. Recruitment flyers were distributed in doctor's offices, psychotherapeutic practices and self‐help organizations for PSS. All participants gave written consent to participate beforehand. The participants received an expense allowance of 20 Euros/hour and were reimbursed for travel costs.

### 2.4 Questionnaires

Participants filled out questionnaires on sociodemographic data. Data on PSS, depression and anxiety severity were collected with the Patient‐Health Questionnaire 15,^27^ the Somatic Symptom Disorder B‐Criteria Scale,^28^ the Stigma‐9 Questionnaire^29^ and the Patient Health Questionnaire‐4.^30^

After each WS, all participants including the PRT evaluated form and structure, characteristics of the moderators as well as the scope and meaning of the WS with items used in a study by Brütt, Meister^31^ on a 5‐point Likert scale (0 = *strongly disagree* to 4 = *strongly agree*). To evaluate the quality of PPI, participants were asked to fill out the German version of the Public and Patient Engagement Evaluation Tool (PPEET).^32^ The PPEET evaluates PPI in research projects in the domains Communication and Support, Views and Perspectives, Engagement Initiative and Final Thoughts on a 5‐point Likert scale (0 = *strongly disagree* to 4 = *strongly agree*). We added four items to evaluate the participants' perception of the collaboration between participants and researchers.

### 2.5 Data analyses

The workshops were recorded and transcribed verbatim. The transcripts were anonymized according to German data protection regulations. We used thematic analysis^33^ to identify common themes. The coding process was based on a deductive approach, to answer previously defined research questions concerning barriers in healthcare, content‐related needs and preferences on design and language. Two independent researchers with a master's degree in psychology (E. F., N. H.) performed the analysis to ensure trustworthiness and rigour. The analysis was performed with MAXQDA.^34^ The generated codes and themes were further discussed with S. K. (PhD Clinical Psychology) to reach consensus. The PPEET^32^ and other items on PPI were analyzed quantitatively regarding frequencies with SPSS 27^35^ to evaluate the participation process.

## 3 RESULTS

### 3.1 Participants

Twelve individuals participated (WS I, *n* = 11; WS II, *n* = nine; WS III, *n* = six; WS IV, *n* = 10). Characteristics of the sample are shown in Table 1. Five were married and seven were single. Seven held an A‐level degree and five had graduated from middle school. Four participants were currently employed, four were retired, two were unemployed, one was in university and one had applied for disability pension. Four were on sick leave at the time of participation. All of the participants reported suffering from PSS for at least 6 months. The self‐reported diagnosis associated with the persistence of somatic symptoms were fibromyalgia, polyneuropathy, fatigue, postexertional malaise, irritable bowel syndrome and chronic pain in different body parts. Eight participants felt they were well informed about PSS. Some had prior experience as research participants, but none of them had engaged in PPI initiatives. Participants reported gaining knowledge, helping others, interest in research and compensation as reasons for their participation. Two participants dropped out after WS I due to time constraints.

**Table 1:** Description of study sample (*N* = 12).

	*M* (SD)	Min–max	*N* (%)
Age	48.3 (14.8)	20–66	
Duration of PSS in months	134.9 (185.5)	10–516	
Gender			
Female			8 (66.7)
Male			4 (33.3)
Diverse			0 (0.0)
Somatic symptom severity (PHQ‐15)	12.3 (4.8)		
Psychological symptom severity (SSD‐12)	28.3 (4.7)		
Mental health‐related stigma beliefs (Stig‐9)	13.7 (4.6)		
Anxiety severity (PHQ‐4)	3.1 (2.3)		
Depression severity (PHQ‐4)	2.4 (1.9)		

Abbreviations: M, mean; Max, maximum; Min, minimum; *N*, number of respondents; PHQ‐4, Patient Health Questionnaire 4; PHQ‐15, Patient Health Questionnaire 15; PSS, persistent somatic symptoms; SD, standard deviation; SSD‐12, Somatic Symptom Disorder B‐Criteria Scale; Stig‐9, The Stigma‐9 Questionnaire.

### 3.2 Thematic analysis

Thematic analysis was conducted to investigate three concepts: (a) perceived barriers in healthcare, (b) participants' needs for GUIDE.PSS and (c) practical implications for GUIDE.PSS. Practical implications included content, language and design of the internet‐based guide.

#### 3.2.1 Perceived barriers in healthcare

Participants reported organizational, practitioner‐related and patient‐related barriers to seeking treatment for PSS. Barriers related to organizational structures included short duration of consultations and shortage of specialized practitioners.

Practitioner‐related barriers included lack of knowledge of PSS: ‘In my case, I got all the information myself, or had to get it myself. Because unfortunately no doctor and no institution knew about this disease’ (P3). They also reported that practitioners failed to adequately introduce them to the psychological components of their symptoms and did not feel validated during consultations.

The majority of participants reported stigma beliefs as a patient‐related barrier. They feared that psychological symptom attribution undermined the legitimacy of their physical experiences. One participant opposed the idea of a psychosomatic disease model: ‘(…) my family doctor classified me as a purely psychological case. But I'm very resilient and I'm not a suffering person and somehow I had to make it clear to him that I really don't have anything mentally. That this is physical. (…) That was pretty difficult’ (P3). Some participants also held stigma beliefs concerning psychotherapy. Many reported resignations after experiencing years of severe symptoms and rather relying on short‐term management strategies, for example, pain medication. Finally, their own lack of knowledge was reported as a contributing factor to long duration of untreated illness. Participants believed that seeking information at an early stage could have helped them in managing their symptoms with more foresight.

#### 3.2.2 Needs for GUIDE.PSS

Participants had unmet needs that GUIDE.PSS should address. Participants most frequently expressed their need to feel more supported and less isolated. Participants also wanted to feel more validated in their concerns: ‘(…) if you don't feel taken seriously, then you go somewhere where you might be taken seriously’ (P1). Several participants wished that the guide would contribute to destigmatizing PSS and the utilization of mental healthcare. Feeling more in control of their illness and more engaged with managing their symptoms were also identified as relevant objectives.

Participants agreed that first impressions and attributes were important to successfully address those needs and engage users. They suggested the guide should make a trustworthy and credible impression. GUIDE.PSS should also be understandable, interesting and arouse curiosity. Several participants stated that the guide needed to be empathic, encouraging and should convey a positive attitude, without raising false hopes.

#### 3.2.3 Practical implications for GUIDE.PSS

##### Content

**Figure 1:**
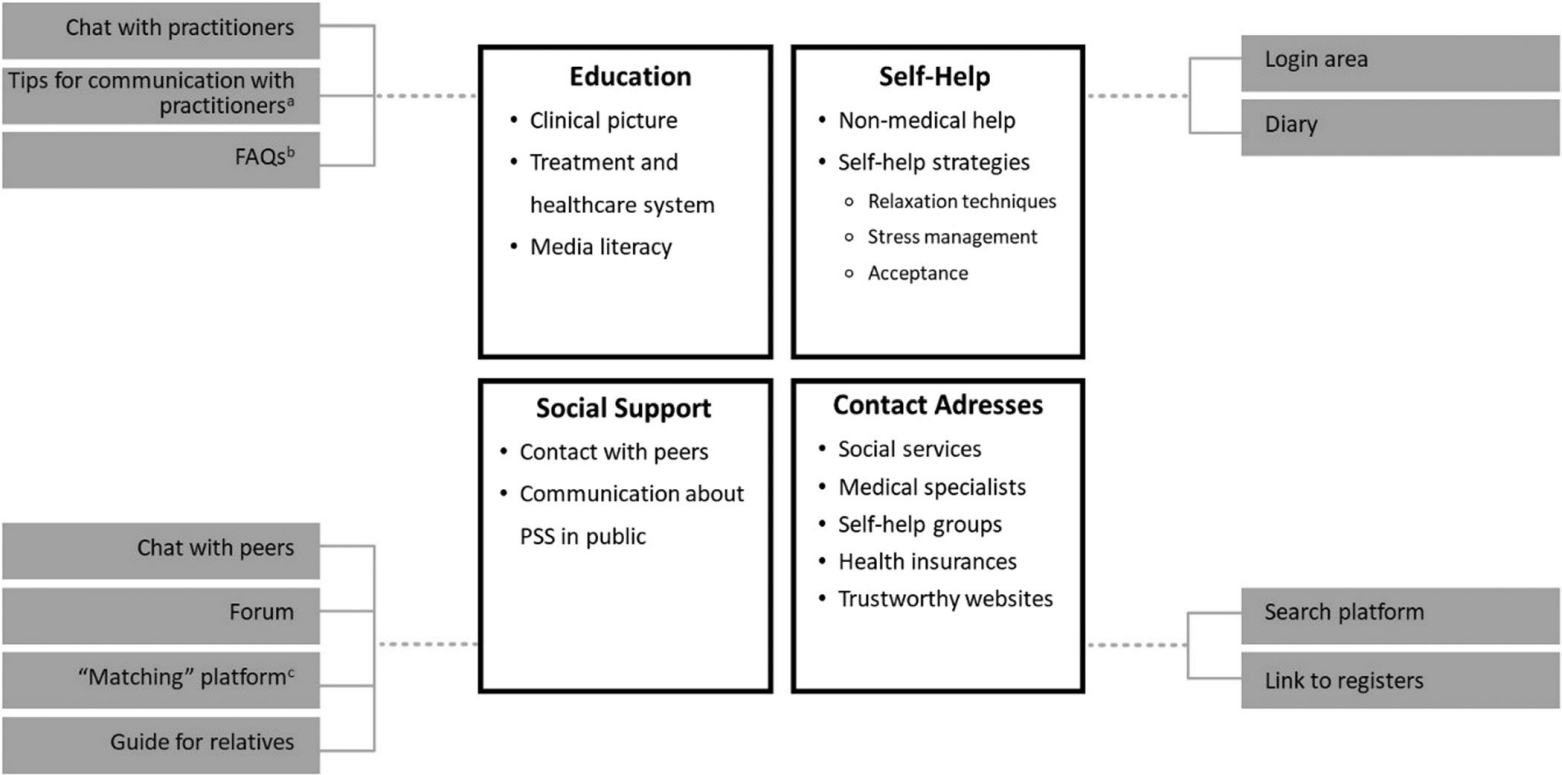
GUIDE.PSS content categories. The figure shows the four main content categories, respective subcontents and features. ^a^Option to download a document with tips on how to communicate symptoms and prepare for consultations. ^b^Brief answers to frequently asked questions concerning PSS for a first overview. ^c^Mobile application where users can match with other profiles that meet their search criteria. FAQs, frequently asked questions; PSS, persistent somatic symptom.

Resulting from participants' needs, four main content categories for GUIDE.PSS were identified: education, self‐help, social support and contact addresses. The contents and respective subcontents are shown in Figure 1.

##### Language

Participants stressed that the language should be motivating and engaging. Participants preferred short and concise paragraphs, figurative language and case studies. Most of them preferred a casual tone, while others felt a formal tone was more appropriate and validating. Several participants believed that biomedical terms were not appropriate for the target audience. Others, however, felt that medical terms lead to more credibility, depending on the respective content. There was no clear consensus on being addressed directly, however, there were concerns that a neutral wording could reduce user engagement: ‘(…) “Here is more information” instead of “Here you can find more information”. That's neutral, nobody feels bad about it’ (P6). ‘(…) but it may come across as very impersonal’ (P5). Many felt the terms ‘psychotherapy’ or ‘psychosomatic’ were too labelling. They preferred nonstigmatizing terminology, for example, ‘expert’ instead of ‘psychotherapist’.

##### Design

Most participants were in favour of an interactive guide. Some participants suggested including a login function to store personal data. They preferred a simple and intuitive design and emphasized the importance of visual material. Some felt that illustrations should reflect the theme of the respective content. Most participants preferred a colourful design. They agreed that the design should indicate PPI during intervention development as a sign of quality and trustworthiness, for example, via emblems: ‘What I would really make bigger is the “Developed by those affected…” emblem. (…) Because that is a mark of quality. It kind of builds trust’ (P15). The preferences concerning other design elements varied between participants.

### 3.3 PPI evaluation

A total of 35 ratings from all WS were analyzed to evaluate PPI (WS I, *n* = 10, WS II, *n* = nine; WS III, *n* = six; WS IV, *n* = 10). Data from one questionnaire was missing. Participants agreed or strongly agreed that the workshop was clearly structured (97.1%), learning goals were clearly defined (94.3%), and moderators were open to criticism (100.0%), encouraged to critically deal with the topics covered (94.3%) and were helpful (100.0%). The majority of participants agreed or strongly agreed that the meaning of topics covered was high (77.1%), the content of the workshops was covered in an adequate pace (88.6%) and the amount of topics were adequate (91.4%).

**Figure 2:**
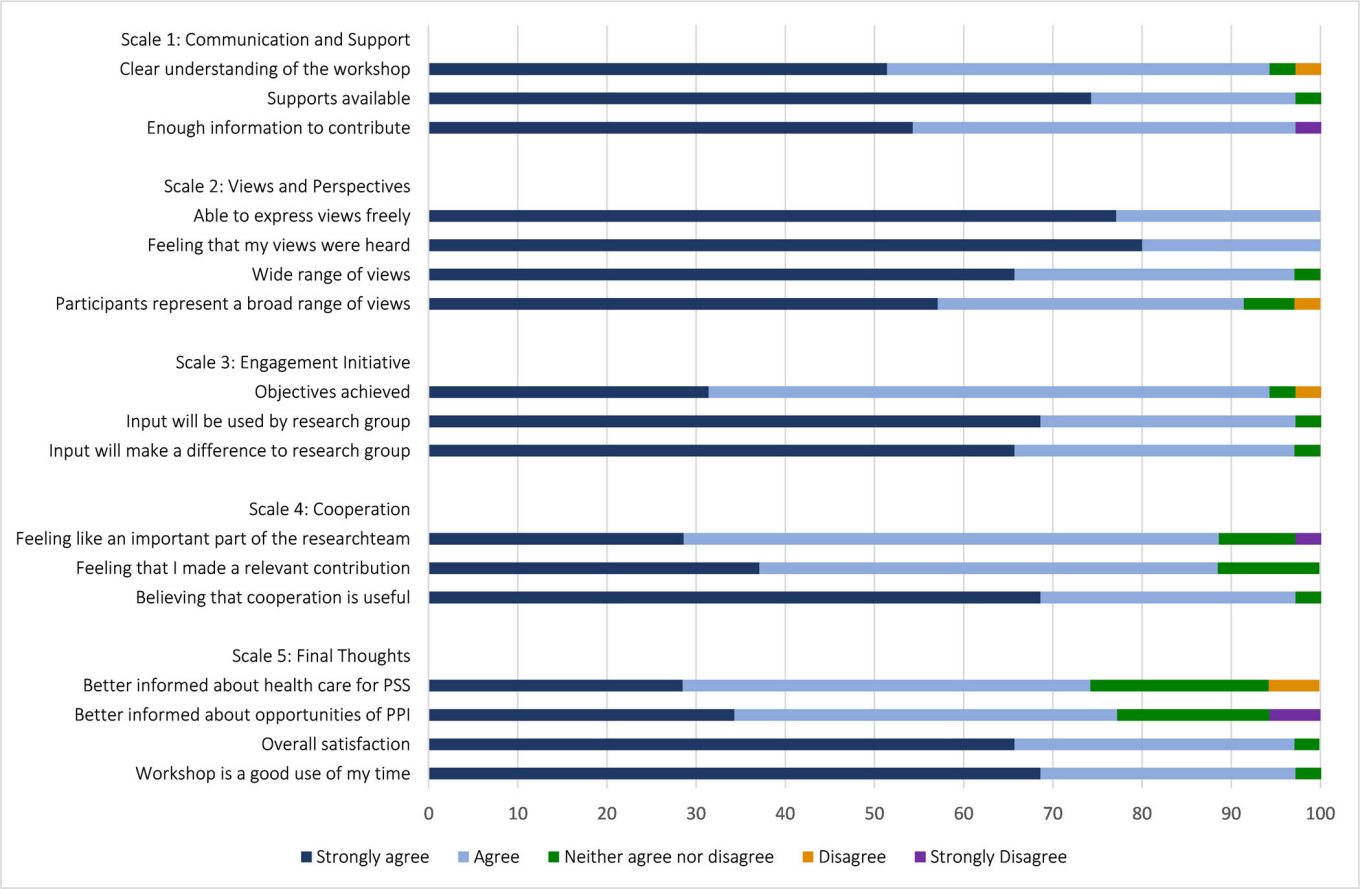
Public and patient engagement evaluation tool. Frequencies are shown in %. Summarized evaluation of all workshops (WS I, *n* = 10; WS II, *n* = 9; WS III, *n* = 6; WS IV, *n* = 10).

The PPEET results showed that the majority of participants agreed or strongly agreed that the workshops achieved their objectives in the domains Communication and Support (≥94.3%), Views and Perspectives (≥91.4%), Engagement Initiative (≥94.3%) and Cooperation (≥88.5%). Several participants stated they appreciated the friendly and accepting atmosphere between participants and researchers. They felt that they were taken seriously and could share their experiences openly. Some participants suggested that more time and a smaller group size would have been beneficial. One participant reported that illness‐related concentration problems negatively affected their ability to engage in the workshop. One participant noted that the different opinions and variations in symptoms were challenging during group tasks. PPEET results are shown in Figure 2.

### 3.4 Impacts of PPI

PPI had a substantial impact on the drafting of the GUIDE.PSS prototype. People with lived experiences made the final decision on the content of GUIDE.PSS. While not all suggestions could be incorporated due to lack of evidence, all content categories were suggested and approved by participants (see Figure 1). While the researchers had not defined any content in advance, the perspective of those affected changed their initial assumptions, especially the importance of social support and the expressed need to feel less alone with one's illness may have been underestimated by researchers.

**Figure 3:**
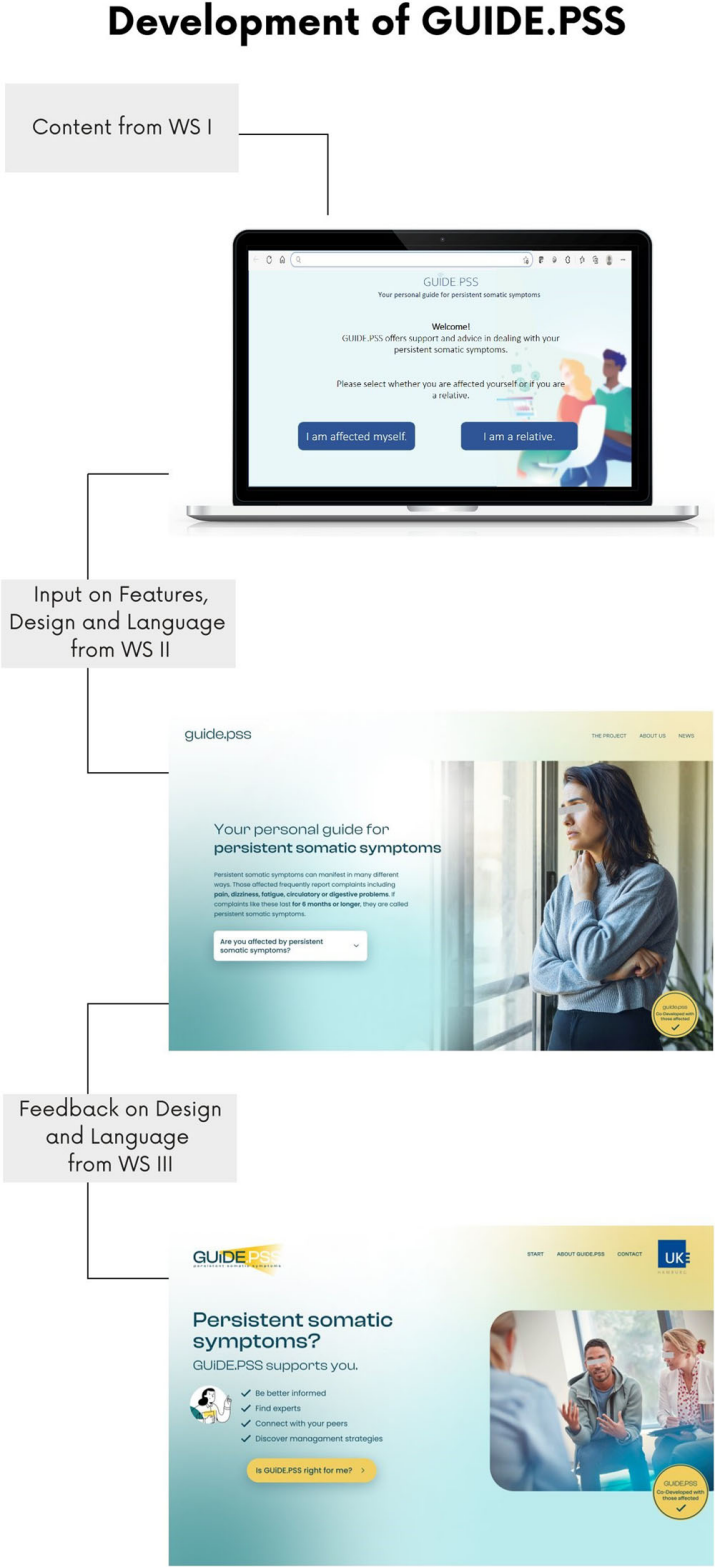
Stages of GUIDE.PSS prototype from WS II to WS IV. The figure shows the stages of the prototype from WS II to WS IV. The development process followed an iterative process with constant feedback loops. The prototypes were translated from German.

Changes in the design of the first page of the prototype are shown in Figure 3. In the initial draft, the landing page included the option to select whether you are affected yourself or whether you are a relative seeking information on PSS. Since participants felt the main focus should be on those affected, the guide for relatives was designed to be less present. Participants also preferred to include a short overview of what to expect from the guide as bullet points, to arouse curiosity and manage expectations. They wanted to include a button with more detailed information on the aim and target population of GUIDE.PSS to avoid confusion. While pictures conveying negative emotions were perceived as demotivating, positive emotions on the other hand made them feel invalidated in their concerns. Hence, they agreed on a fairly neutral picture, depicting a consultation with a practitioner. The level of contribution through PPI ranged from major decisions, for example, content, to detailed decisions about design, for example, the GUIDE.PSS logo. The results described in Section 3.2 were also incorporated into the prototype.

## 4 DISCUSSION

The aim was to identify needs of those affected by PSS for an internet‐based guide and to evaluate impact, process and outcome of PPI. PPI contributed to the identification of main features, content and design of the internet‐based guide. People with lived experiences wanted to feel more supported, validated, in control and engaged with managing their health. Content‐related needs included education, self‐help, social support and contact addresses. They preferred a tailored and interactive guide with visual material. Preferences on the tone and terminology varied. The development followed an iterative process with feedback loops to incorporate those needs. Some preferences, including nonevidence‐based alternative treatments, were not incorporated into the prototype to ensure evidence‐based content. Participants expressed overall satisfaction with the PPI process, highlighting the perceived value of involving stakeholders in research projects.

The content‐related needs are consistent with previous studies on PSS.^18^ Participants reported feelings of isolation and stigma beliefs as major challenges, which is in line with previous reports.^18^, ^19^, ^36^ There was an expressed need for validation of their illness. This was partially in conflict with the concept of a biopsychosocial disease model, as those affected often assume that their symptoms stem from severe physical illnesses.^19^ During the workshops, the construct of ‘PSS’ was perceived as understandable and acceptable. Participants expressed the desire to feel more in control of their illness, which aligns with other reports on the challenges of dealing with uncertainty in PSS.^18^ Participants also highlighted the importance of feeling more engaged in managing their symptoms, as many had experienced resignation as a maladaptive coping strategy. Similar to other reports, lack of knowledge was also identified as a contributing factor to prolonged search for treatment.^18^

Results regarding design and language mostly align with recommendations for patient education material, including short and concise paragraphs and visual material that reinforces the content.^37^ Consistent with other studies, participants favoured a tailored programme,^38^ as it enhances personal relevance. Participants preferred the option to contact practitioners directly, indicating the significance of guidance from general practitioners when using internet‐based programmes.^18^ Interestingly, some participants favoured medical terminology, which is in conflict with previous studies on the general population.^39^ Medical terminology may reassure those affected by PSS in assumptions of a biomedical disease model.^19^ This was also reflected in the perception of the terms ‘psychosomatic’ and ‘psychotherapy’ as too labelling, which is consistent with other studies on PSS.^40^ This emphasizes the importance of cautiously introducing psychological components, while validating the physical experience of those affected. There was a lack of consensus on using a casual or formal tone. A study on users' preferences for an intervention to promote oral human immunodeficiency virus self‐testing found that preferences on tone depended on the respective content.^41^ Hence, it may be useful to address motivational topics in a more casual way than educational topics. Further research is necessary to clarify preferences on language.

Our findings demonstrate the benefits of involving people with lived experiences in the early development of novel interventions. The involvement played a crucial role in identifying and incorporating needs of those affected by PSS in the design of an internet‐based guide. Participants identified the content of the novel intervention and assessed the appropriateness of language and design. They prioritized outcomes for the follow‐up study, which is important to ensure relevance for the target population.^42^ Overall, PPI had a significant impact on the development of the intervention and broadened the perspective of the research team. Participants reported a comfortable group atmosphere, were able to express their views freely and felt that they were heard. This may be especially valuable within a group of affected individuals that often experience stigma and lack of validation within the health care system.^19^ There was strong agreement that their input made a difference to the research group, which is an important factor of success of PPI,^13^ as participants often feel PPI is merely tokenistic.^43^ Participants were convinced that PPI contributed to the quality and trustworthiness of the internet‐based guide. This outcome supports the notion that PPI, when properly implemented, can contribute to the development of interventions tailored to the specific needs of the target population.

While PPI made a valuable contribution to the development process, there were some complications. Researchers had predetermined that the guide should refer to recommendations from the current German guidelines to ensure evidence‐based content.^24^ This decision sometimes conflicted with participants' perspectives, especially concerning alternative treatment options. This highlights the challenge of balancing evidence‐based practices with the perspectives of people with lived experiences. Additionally, limitations in funding and time constrained the implementation of certain features of the guide participants' favoured, highlighting the importance of considering resource limitations when planning PPI initiatives. Another complication was the lack of consensus among participants on certain topics. Dominant individuals may have overpowered other participants, which is a common challenge in group settings.^13^ This emphasizes the importance of facilitating balanced participation and ensuring that all voices are heard equally. Misaligned expectations were another complication encountered. Although researchers clarified the projects' scope and aim in the initial workshop, some participants expected access to the final guide, which could not be fulfilled due to practical constraints. Continuously aligning expectations with participants may help manage their expectations and prevent misunderstandings. A few participants did not feel better informed about opportunities for PPI after the project. Providing clear and accessible information to the general public about how individuals can get involved in research can help increase participation and engagement in PPI initiatives in the future.

### 4.1 Limitations

First, due to the qualitative nature of the study, the results cannot be generalized, but the sample characteristics were diverse concerning lived experiences with PSS. Second, as there are age‐related effects on the use of internet‐based mental health interventions,^38^ it is possible that a younger sample would have offered a different perspective. However, due to the recruitment strategy, participants still represented a broad range of symptoms and experiences with PSS, and there were two participants under the age of 30. Third, it is possible that not all topics were equally addressed due to limitations in time. Fourth, participation varied over the course of the study and two participants dropped out after the first workshop due to time constraints. Finally, it should be noted that this paper reports the development stage of a novel intervention that needs to be further evaluated in follow‐up studies.

## 5 CONCLUSION

To the best of our knowledge, this is one of the first studies informing the development of an internet‐based guide for PSS with principles of PPI. Those affected by PSS are often viewed as a burden in health care settings,^19^ but our study demonstrates the value of involving them as active research partners. PPI in early intervention development allowed to acknowledge their unheard perspective and ensure the development of an intervention that is tailored to the needs of the target population. The benefits of PPI should receive more attention within PSS research to successfully address a previously unreached target population. Nevertheless, more evidence‐based studies are needed to assess the effects of PPI on intervention development. The involvement of people with lived experiences revealed several needs of those affected by PSS that should be taken into consideration when designing interventions to address the existing treatment gap. The identified needs and preferences concerning content, language and design were incorporated in the development of the GUIDE.PSS prototype. The extent to which GUIDE.PSS is rated as helpful, informative and supportive and will be evaluated in a follow‐up study using experimental designs.

## AUTHOR CONTRIBUTIONS

Sebastian Kohlmann designed the study. Bernd Löwe and Sebastian Kohlmann obtained the funding. Eirin Fränkl, Nele Hasenbank and Sebastian Kohlmann collected the data and performed data analysis. Karin Dumröse was involved in data collection and checked data interpretation. Eirin Fränkl wrote the original draft of the manuscript under the supervision of Sebastian Kohlmann. All authors critically reviewed and edited the original draft. All authors had full access to all the data in the study, approved the final version of the manuscript, and took responsibility for its submission for publication.

## CONFLICT OF INTEREST STATEMENT

The authors declare no conflict of interest.

## ETHICS STATEMENT

This study was approved by the local Ethics committee at the Centre for Psychosocial Medicine (# LPEK‐0554). Written informed consent for the publication of their details was obtained from the patients.

## Data Availability

The data that support the findings of this study are available from the corresponding author upon reasonable request.
